# Body weight prediction using body size measurements in Fleckvieh, Holstein, and Brown Swiss dairy cows in lactation and dry periods

**DOI:** 10.5194/aab-61-413-2018

**Published:** 2018-10-30

**Authors:** Leonhard Gruber, Maria Ledinek, Franz Steininger, Birgit Fuerst-Waltl, Karl Zottl, Martin Royer, Kurt Krimberger, Martin Mayerhofer, Christa Egger-Danner

**Affiliations:** 1 Agricultural Research and Education Centre Raumberg-Gumpenstein, Irdning-Donnersbachtal, 8952, Austria; 2 Department of Sustainable Agricultural Systems, BOKU – University of Natural Resources and Life Sciences Vienna, Vienna, 1180, Austria; 3 ZuchtData EDV-Dienstleistungen GmbH, Vienna, 1200, Austria; 4 LKV Niederösterreich, Zwettl, 3910, Austria; * These authors contributed equally to this work

## Abstract

The objective of this study was to predict cows' body weight from body size measurements and other animal data in the lactation and dry periods. During the whole year 2014, 6306 cows (on 167 commercial Austrian dairy farms) were weighed at each routine performance recording and body size measurements like heart girth (HG), belly girth (BG), and body condition score (BCS) were recorded. Data on linear traits like hip width (HW), stature, and body depth were collected three times a year. Cows belonged to the genotypes Fleckvieh (and Red Holstein crosses), Holstein, and Brown Swiss. Body measurements were tested as single predictors and in multiple regressions according to their prediction accuracy and their correlations with body weight. For validation, data sets were split randomly into independent subsets for estimation and validation. Within the prediction models with a single body measurement, heart girth influenced relationship with body weight most, with a lowest root mean square error (RMSE) of 39.0 kg, followed by belly girth (39.3 kg) and hip width (49.9 kg). All other body measurements and BCS resulted in a RMSE of higher than 50.0 kg. The model with heart and belly girth (Model${}_{\mathrm{HG}\;\mathrm{BG}}$) reduced RMSE to 32.5 kg, and adding HW reduced it further to 30.4 kg (Model${}_{\mathrm{HG}\;\mathrm{BG}\;\mathrm{HW}}$). As RMSE and the coefficient of determination improved, genotype-specific regression coefficients for body measurements were introduced in addition to the pooled ones. The most accurate equations, Model${}_{\mathrm{HG}\;\mathrm{BG}}$ and Model${}_{\mathrm{HG}\;\mathrm{BG}\;\mathrm{HW}}$, were validated separately for the lactation and dry periods. Root mean square prediction error (RMSPE) ranged between 36.5 and 37.0 kg (Model${}_{\mathrm{HG}\;\mathrm{BG}\;\mathrm{HW}}$, Model${}_{\mathrm{HG}\;\mathrm{BG}}$, lactation) and 39.9 and 41.3 kg (Model${}_{\mathrm{HG}\;\mathrm{BG}\;\mathrm{HW}}$, Model${}_{\mathrm{HG}\;\mathrm{BG}}$, dry period). Accuracy of the predictions was evaluated by decomposing the mean square prediction error (MSPE) into error due to central tendency, error due to regression, and error due to disturbance. On average, 99.6 % of the variance between estimated and observed values was caused by disturbance, meaning that predictions were valid and without systematic estimation error. On the one hand, this indicates that the chosen traits sufficiently depicted factors influencing body weight. On the other hand, the data set was very heterogeneous and large. To ensure high prediction accuracy, it was necessary to include body girth traits for body weight estimation.

## 1 Introduction

There are various reasons for predicting the body weight of dairy cows and a number of different ways to do so. Heinrichs et al. (1992) developed prediction models to facilitate a better understanding of heifer growth or treatments on growth. They regressed body weight based on heart girth, height at withers, hip width, or body length. Enevoldsen and Kristensen (1997) estimated body weight by combining the previously rarely used body measurements hip height, hip width, and body condition score (BCS) and considering other animal-specific information like parity and day in milk (DIM). In the UK, linear conformation traits have previously been used to predict body weight (Koenen and Groen, 1998; Coffey et al., 2003) and for management purposes (Coffey et al., 2003). As the database was from the 1990s, Banos and Coffey (2012) updated their prediction model on the phenotypic and genetic level, finally resulting in a combination of stature, chest width, body depth, and angularity. Similarly, Heinrichs et al. (2017) reviewed their previously developed prediction model and found it to be sufficient. Yan et al. (2009) used data from 146 cows of a research herd to predict body weight and empty body composition. Haile-Mariam et al. (2014) predicted body weight from linear conformation traits of 430 000 Australian Holstein (HF) cows and examined the relationship between body weight and production and fitness traits. In recent decades, the scientific importance of body weight has increased due to its connection to feed intake and efficiency traits. Body weight or related traits have been included in the (national) breeding indices of Holstein US, New Zealand, and Australia, and spring-calving herds in the UK (VanRaden, 2004; Harris et al., 2007; Haile-Mariam et al., 2013; Mike Coffey, personal communication, 2017).

With the exception of the studies by Enevoldsen and Kristensen (1997) and Haile-Mariam et al. (2014), predictions of cow body weight have until now been derived from research herds, and the numbers of cows or observations were mostly low. Due to lack of data, validation had to be made internally by additionally splitting these relatively small data sets. Prediction models based on small data sets tend to be very accurate within this data set, but validity for the whole population can be low. Furthermore, while prediction models including body girth measurements or BCS were more accurate than those based solely on linear traits, no incidence of a routine recording traits other than linear traits could be found in the literature. Another point is that body weight has previously been predicted for lactating cows only, although both body weight and body condition are crucial parameters at the end of gestation and thus during the dry period. The physical constitution at calving and management in the dry period indirectly influences production, health, and fertility in the subsequent lactation (Roche et al., 2009).

The objective of this study was to develop and evaluate body weight prediction models for dairy cows during the whole production cycle. In addition to routinely measured linear traits like stature, body depth, or hip width, BCS and muscle score as well as novel traits like heart and belly girth were investigated to take advantage of their high prediction accuracy.

## 2 Materials and methods

### 2.1 Data recording and database

Data obtained were based on a 1-year data collection period in 2014 and taken from a total of 3750 Fleckvieh (FV), 1056 Holstein-Friesian (HF), and 1500 Brown Swiss (BS) cows kept on 167 Austrian dairy farms (44 441 recordings). The majority of the cows was milked twice a day in a milking parlor system and kept in free-stall barns. In addition to routine performance recording, the Austrian milk recording organizations weighed both lactating and dry cows using a mobile scale and collected several body measurements. Data were entered in the Austrian central cattle database. The following four body traits were recorded at each routine performance recording (nearly each month, up to 12 times per year). Heart girth (HG): tape measure, behind shoulder around cow; belly girth (BG): tape measure, around belly just in front of the udder; body condition score (BCS): five-point system by Edmonson et al. (1989); and muscle score (MUSC): points 1–10 (1 = poorly to 10 = highly muscled).

The following seven linear traits were measured up to three times per cow with a measuring stick by official classifiers of the Federation of Austrian Cattle Breeders. Stature (ST): measured from the top of the spine in between hips to ground; body length (BL): distance between withers height and at the top of the spine between the hips; pelvis length (PL): distance between pin bones and hip bones; body depth (BD): distance between top of spine and bottom of barrel at last rib – the deepest point, independent of stature; hip width (HW): distance between hip bones; pin width (PW): distance between pin bones; and knee width (KW): distance between the knees. As several different measuring points for defining body width are internationally in common use, hip width, pin width, and knee width were named after their specific anatomic characteristics to avoid any possibility of confusion.

**Table 1:** Description of the estimation and validation subset for Model${}_{\mathrm{HG}\;\mathrm{BG}\;\mathrm{HW}}$ during lactation (HG: heart girth; BG: belly girth; HW: hip width).

Trait	Estimation subset							Validation subset					
	n	Mean	Standard	Variation	Minimum	Maximum		n	Mean	Standard	Variation	Minimum	Maximum
			deviation	coefficient						deviation	coefficient		
Body weight, kg	8,474	702	88.9	12.7	416	1016		2080	702	90.1	12.8	435	1013
Body weight estimated, kg								2080	702	80.8	11.5	441	980
Heart girth, cm	8474	210	10.3	4.9	173	253		2080	210	10.3	4.9	175	247
Belly girth, cm	8474	256	13.6	5.3	204	312		2080	256	13.9	5.4	199	298
Stature, cm	8473	146	4.5	3.1	128	163		2080	146	4.7	3.2	130	163
Body length, cm	7947	90	5.6	6.2	73	111		1969	91	5.6	6.2	74	109
Pelvis length, cm	8469	56	3.0	5.4	46	68		2080	56	3.0	5.4	45	66
Body depth, cm	8471	84	4.5	5.4	67	99		2077	84	4.5	5.4	69	99
Hip width, cm	8474	57	3.4	5.9	45	68		2080	57	3.4	6.0	46	68
Pin width, cm	8435	39	4.7	12.0	27	59		2073	39	4.9	12.6	27	59
Knee width, cm	8427	53	5.5	10.3	36	69		2072	53	5.6	10.4	34	71
BCS, points 1–5	8414	3.15	0.58	18.6	1.00	5.00		2065	3.15	0.57	18.1	1.00	5.00
Muscle score, points 1–10	8419	5.1	1.5	29.4	1.0	9.0		2067	5.1	1.5	28.9	1.0	9.0
Parity	8474	3.0	2.0	65.8	1	13		2080	3.0	2.0	66.5	1	13
Day relative to calving	8474	160	96	60	1	364		2080	164	97	59	1	364

Model${}_{\mathrm{HG}\;\mathrm{BG}}$ has similar means and standard deviation despite the higher number of animals.

**Table 2:** Description of the estimation and validation subset for Model${}_{\mathrm{HG}\;\mathrm{BG}\;\mathrm{HW}}$ during the dry period (HG: heart girth; BG: belly girth; HW: hip width).

Trait	Estimation subset							Validation subset					
	n	Mean	Standard	Variation	Minimum	Maximum		n	Mean	Standard	Variation	Minimum	Maximum
			deviation	coefficient						deviation	coefficient		
Body weight, kg	909	788	93.6	11.9	528	1071		262	792	89.0	11.2	578	1018
Body weight estimated, kg								262	797	77.7	9.7	609	1046
Heart girth, cm	909	217	11.2	5.1	185	264		262	218	11.0	5.0	190	250
Belly girth, cm	909	271	13.5	5.0	235	307		262	272	13.1	4.8	234	314
Stature, cm	909	146	4.6	3.2	130	163		262	146	4.7	3.2	132	158
Body length, cm	848	91	5.8	6.4	75	109		243	92	5.6	6.1	75	106
Pelvis length, cm	909	56	3.1	5.5	45	66		262	56	2.9	5.1	48	63
Body depth, cm	908	86	4.2	4.8	71	99		262	87	4.4	5.0	75	99
Hip width, cm	909	58	3.4	5.8	47	67		262	58	3.3	5.7	49	70
Pin width, cm	906	40	4.8	12.1	27	57		261	40	5.2	13.0	28	57
Knee width, cm	905	55	6.2	11.2	36	71		261	55	5.8	10.6	38	70
BCS, points 1–5	903	3.57	0.56	15.7	2.00	5.00		262	3.58	0.60	16.8	1.75	5.00
Muscle score, points 1–10	900	5.9	1.6	26.7	1.0	9.0		261	5.9	1.5	25.6	2.0	9.0
Parity	909	2.8	1.8	65.7	1	12		262	2.9	1.7	59.6	1	8
Day relative to calving	909	-26	14	-53	-56	-1		262	-28	14	-49	-56	-1

Model${}_{\mathrm{HG}\;\mathrm{BG}}$ has similar means and standard deviation despite the higher number of animals.

On average, cows weighed 699 kg during lactation, and were approximately 95 kg heavier during the dry period (Tables 1, 2, S1, and S2). Dry cows had a wider heart and belly girth, while other linear body measurements changed only slightly. The data set was characterized by a high variation between animals in body size measurements, body weight, and BCS. Body weight ranged between 400 and 1088 kg during lactation and between 506 and 1108 kg during the dry period. Minimum and maximum heart girths were 166 and 257 cm during lactation. Overall stature ranged between 128 and 163 cm. Therefore the body weight prediction models were based on a relatively large data set including a high diversity of individuals.

### 2.2 Statistical analysis

The fixed effects of the basic model (Eq. 1) were defined in a series of preliminary tests. In accordance with Banos and Coffey (2012), factors with the highest significant influence on body weight were included.

The classes HF and BS of the fixed effect genotype included cows with 100 % HF and BS ancestry. Fleckvieh cows were classified into several groups according to their Red Holstein (RH) gene proportion. This classification was chosen in accordance with the results of Ledinek et al. (2018). The number of classes was reduced for easier handling. In the FV class, the two groups with 100 % FV and an average of 6.25 % RH ancestry were combined due to a lack of significant differences and included 2604 cows with ≤10 % RH genes. In FV × RH_m, the two FV × RH groups with an average of 12.5 and 25 % RH genes were combined (>10 to ≤44.5 %, medium proportion of RH genes, 773 cows). Cows with a high proportion of RH were included in FV × RH_h (FV with >44.5 % genes, 373 cows). Genotypes were analyzed together to characterize the influence of genotype on body weight, as HF and BS are specialized dairy types and FV is a dual-purpose breed. Additionally, this enables the identification of a possible genotype-specific influence of body measurements on body weight.

The fixed effect physiological stage combined the lactation stage with 13 months each (days in milk – DIM – 1 to 364, 28 days per month) and the dry period with four 2-week stages (days -56 to -1 relative to calving). Preliminary tests had shown that prediction accuracy profited noticeably from combining the lactation and dry periods. The fixed effect of parity consisted of the classes 1, 2, 3+4, and ≥5.

The SAS 9.4 software package (SAS, 2015) was used for statistical analysis. PROC MIXED and the REML method (estimation of variance components) were chosen. The Kenward–Roger method was applied to approximate the denominator degrees of freedom as well as the covariance structure VC, which caused the smallest Akaike information criterion. The following basic model was used for testing body measurements (Eq. 1):

1 $$Y_{ijklm}=\mu +G_{i}+P_{j}+{\mathrm{PS}}_{k}+\sum b_{l}\times X_{l}+F_{m}+\varepsilon_{ijklm},$$

where $Y_{ijklm}$ is observed body weight, μ is the intercept, $G_{i}$ is the fixed effect of genotype, $P_{j}$ is the fixed effect of parity, PS${}_{k}$ is the fixed effect of physiological stage (lactation and dry periods), $b_{l}$ is the linear regression on the lth body measurement ($X_{l}$) summed over all body measurements, and $F_{m}$ is the random effect of farm (m=1–167); $\varepsilon_{ijklm}$ is the residual. Although various approaches were tested, matrices of models that included the effect of cow were not calculable. But it should be pointed out that estimates and estimation errors were well within the common range as reported in the literature.

First, each body measurement was tested within the basic model as a single predictor in linear and quadratic regressions (Eq. 1). Then, further body measurements were added in multiple linear regressions according to their quality of estimation and their Pearson correlations (r) related to body weight. Similar approaches were chosen in other studies (Yan et al., 2009; Banos and Coffey, 2012; Haile-Mariam et al., 2014). The influence of body measurements was evaluated according to the Akaike information criterion, the root mean square error (RMSE), the significance of model parameters, and the impact of model parameters on least squares means (LSMs) and regression coefficients. The two most accurate equations, Model${}_{\mathrm{HG}\;\mathrm{BG}}$ and Model${}_{\mathrm{HG}\;\mathrm{BG}\;\mathrm{HW}}$, were chosen for validation and used to create the final advanced models (Eq. 2). The data set of the respective model was therefore randomly split into a single estimation and a single validation subset with 80 and 20 % of the data (Tables 1 and 2 Model${}_{\mathrm{HG}\;\mathrm{BG}\;\mathrm{HW}}$, Tables S1 and S2 in the Supplement Model${}_{\mathrm{HG}\;\mathrm{BG}}$), respectively, including the lactation and dry periods. Each fifth data record was selected for validation. The data set was sorted by the cows' national identification numbers. This resulted in an even distribution within the classes of fixed effects and prevented any trend within traits. The estimation subset of Model${}_{\mathrm{HG}\;\mathrm{BG}}$ contained an average of 5.7 measurements per cow and an average of 37.2 cows per farm, ranging between 2 and 115 cows (validation subset: 1.4 measurements; 33.2, 2–100 cows). The average number of measurements per cow was 1.9 and 1.0 in the estimation and validation subset of Model${}_{\mathrm{HG}\;\mathrm{BG}\;\mathrm{HW}}$. The mean number of cows per farm was 31.4 (2–84 cows) and 14.5 (1–47 cows) in both subsets. Tables S3 and S4 present the number of data records within the classes of the fixed effects of the subsets. The testing of interactions between model parameters identified the strong influence of genotype × body measurement on body weight as well as a significant improvement of estimation accuracy. To take advantage of this, genotype-specific regression coefficients $b_{l}(G_{i})$ were tested stepwise for each body measurement (Eq. 2):

Yijklm=μ+Gi+Pj+PSk+∑bl×Xl+∑blGi2×Xl+Fm+εijklm,

where $b_{l}(G_{i})$ is the linear regression on the lth body measurement ($X_{l}$) for genotype i summed over all body measurements.

As tested in previous studies (Gruber et al., 2004; Ledinek and Gruber, 2015), curves were fitted to the LSMs of the fixed effect of physiological stage separately for the lactation and dry periods. This enables a continuous estimation of body weight depending on DIM and day relative to calving in the dry period.

**Table 3:** Pearson correlation coefficients between body weight, body measurements, body condition score (BCS), and muscle score (MUSC), separated for lactation (above diagonal) and dry (below diagonal) periods.

Trait	BW	HG	BG	ST	BL	PL	BD	HW	PW	KW	BCS	MUSC
BW		0.82	0.82	0.17	0.22	0.41	0.52	0.59	0.43	0.29	0.43	0.46
HG	0.80		0.72	0.25	0.16	0.44	0.55	0.57	0.34	0.21	0.35	0.34
BG	0.80	0.72		0.16	0.25	0.32	0.62	0.54	0.34	0.24	0.30	0.34
ST	0.21	0.25	0.20		0.40	0.50	0.41	0.36	0.04	0.14	0.00${}^{*}$	-0.12
BL	0.15	0.08	0.19	0.43		0.24	0.33	0.32	0.00${}^{*}$	0.05	0.00${}^{*}$	0.01${}^{*}$
PL	0.43	0.40	0.33	0.48	0.23		0.48	0.56	0.23	0.20	0.13	0.02
BD	0.54	0.53	0.60	0.41	0.28	0.46		0.60	0.22	0.24	0.06	0.03
HW	0.57	0.53	0.51	0.34	0.25	0.53	0.54		0.36	0.32	0.16	0.11
PW	0.39	0.29	0.26	0.04${}^{*}$	$-0.06^{*}$	0.22	0.19	0.36		0.21	0.11	0.22
KW	0.25	0.15	0.22	0.19	0.13	0.18	0.27	0.34	0.18		0.22	0.13
BCS	0.48	0.45	0.39	0.04${}^{*}$	0.05${}^{*}$	0.21	0.21	0.27	0.16	0.23		0.58
MUSC	0.45	0.37	0.35	-0.08	0.10	0.10	0.16	0.20	0.17	0.14	0.49	

${}^{*}$ P-value > 0.05; HG: heart girth; BG: belly girth; ST: stature; BL: body length; PL: pelvis length; BD: body depth; HW: hip width; PW: pin width; KW: knee width; MUSC: muscle score.

### 2.3 Methods of testing prediction accuracy

Observed body weight was compared with the predicted body weight in the validation subsets (Tables 1, 2, S1, and S2). The two equations Model${}_{\mathrm{HG}\;\mathrm{BG}}$ and Model${}_{\mathrm{HG}\;\mathrm{BG}\;\mathrm{HW}}$ included the fitted curves for the fixed effect of physiological stage. Models were evaluated separately for the lactation and dry periods. The validation was done according to Bibby and Toutenburg (1977). Bibby and Toutenburg (1977) defined three causes of variance in the deviation of observed to predicted values within the mean square prediction error (MSPE): errors caused by central tendency (ECT), errors due to regression (ER), and errors caused by disturbance (ED). ECT is the difference between the observed and predicted means of body weight and describes a systematic and even under- or over-estimation in the whole body weight range. ER is 0 if the regression coefficient of the linear relationship between observed and predicted values is 1. ECT and ER represent systematic errors and are undesirable. The linear correction of the models can reduce ECT and ER to 0, while ED cannot be reduced (Bibby and Toutenburg, 1977).

To increase the visibility of these possible sources of error, we pictured the predicted values centered around their mean (estimated values minus the mean of estimated values) on the x axis (St.-Pierre, 2003). The differences between observed and predicted values (residuals) were plotted on the y axis.

## 3 Results and discussion

### 3.1 Relationships between body measurements

The accurate prediction of body weight requires body measurements, which can be easily and accurately measured on commercial dairy farms during routine linear scoring, and which enable an accurate prediction. Table 3 shows the Pearson correlations between body measurements separately for the lactation and dry periods. All body measurements correlated positively with body weight. Heart girth and belly girth both had the same and strongest relationship with body weight (r=0.82, 0.80; lactation and dry periods), followed by hip width (r=0.59, 0.57) and body depth (r=0.52, 0.54). The correlations of body weight with heart and belly girth are in agreement with earlier studies reporting correlations between 0.81 and 0.88 (Yan et al., 2009; Ledinek and Gruber, 2014; Stegfellner, 2014). Yan et al. (2009) explained this with the strong connection of body girth measurements to BCS, which agrees with the findings in the current study. Enevoldsen and Kristensen (1997) found a significantly stronger correlation between hip width and body weight (r=0.72) obtained from cows on commercial Danish dairy farms.

In the current study, knee width was additionally recorded in the linear description, to examine its influence on body weight due to its stronger relationship with BCS, as shown in previous Austrian studies (Ledinek and Gruber, 2014; Stegfellner, 2014). In these studies, a strong correlation with body weight ranging from 0.60 to 0.77 was observed. The noticeably lower correlation (r=0.29) in the current study indicates the difficulty of finding the correct points for measuring this novel trait. Hip and pin bones were characterized by an abundant fat layer and therefore showed a lower connection to BCS as compared to the knees during lactation. Therefore hip width, pin width, and pelvis length were easier to measure. Otto et al. (1991) examined hip width and pelvis length and found only very slightly positive relationships with BCS and with the composition of the 9th to 10th rib tissue. Furthermore, in the previous Austrian studies, classifiers were research technicians and had years of prior experience in recording knee width.

**Table 4:** Estimates for the intercept, the fixed effects genotype, parity, and the regression coefficients for the body measurements heart girth (HG), belly girth (BG), and hip width (HW) in the two body weight prediction models Model${}_{\mathrm{HG}\;\mathrm{BG}}$ and Model${}_{\mathrm{HG}\;\mathrm{BG}\;\mathrm{HW}}$.

Estimates	Model${}_{\mathrm{HG}\;\mathrm{BG}}$	Model${}_{\mathrm{HG}\;\mathrm{BG}\;\mathrm{HW}}$
Intercept, kg	-724.81	-833.39
Genotype${}^{1}$, kg		
FV	-101.07	-79.985
FV × RH_m	-121.05	-133.55
FV × RH_h	-148.33	-121.77
HF	-16.418	0.7205
BS	0	0
Parity, kg		
1	-16.797	-9.4629
2	-7.0643	-4.6182
3+4	-0.9199	-2.2144
≥5	0	0
Body measurements${}^{2}$		
Heart girth (HG), kg cm${}^{-1}$		
b_HG	3.1643	2.5192
b_HG × genotype${}^{3}$		
b_HG × FV	1.0631	0.9442
b_HG × FV × RH_m	1.1516	1.2656
b_HG × FV × RH_h	0.7160	0.8393
b_HG × HF	-0.0485	0.00328
b_HG × BS	0	0
Belly girth (BG), kg cm${}^{-1}$		
b_BG	2.9949	2.9030
b_BG × genotype${}^{3}$		
b_BG × FV	-0.3853	-0.4459
b_BG × FV × RH_m	-0.3985	-0.4750
b_BG × FV × RH_h	-0.01813	-0.0904
b_BG × HF	0.00439	0.03483
b_BG × BS	0	0
Hip width (HW), kg cm${}^{-1}$		
b_HW		4.7367
b_HW × genotype${}^{3}$		
b_HW × FV		0.3378
b_HW × FV × RH_m		0.1046
b_HW × FV × RH_h		-0.6885
b_HF × HF		-0.6804
b_HW × BS		0
Physiological stage${}^{4}$, kg		
lactating (DIM = 1 to 365 p.p. – post partum)	2.7426–0.324907 × DIM +	-0.390321-0.00278743 × DIM +
	0.00231406 × DIM${}^{2}-$	0.00231406 × DIM${}^{2}-$
	0.00000567999 × DIM${}^{3}+$	0.00000738974 × DIM${}^{3}+$
	$4.74719\times 10^{-9}$ × DIM${}^{4}$	$7.23071\times 10^{-9}$ × DIM${}^{4}$
dry (DIM = -56 to -1 a.p. – ante partum)	18.755+0.254644 × DIM	17.2602+0.226024 × DIM

${}^{1}$ FV: Fleckvieh with Red Holstein (RH) proportion up to 10.0 %; FV × RH_m: FV with medium RH gene proportion > 10.0 to ≤44.5 %; FV × RH_h: FV with high RH gene proportion > 44.5 %; HF: Holstein Friesian; BS: Brown Swiss. ${}^{2}$ b_: linear regression coefficient. ${}^{3}$ Select the regression coefficient of the respective genotype in addition to the pooled one (b_HG, b_BG, b_HW). ${}^{4}$ Curve fitted to the fixed effect of physiological stage separately for lactation and dry periods.

In accordance with other studies (e.g., Enevoldsen and Kristensen, 1997; Yan et al., 2009; Ledinek and Gruber, 2014), height and length measurements had relatively little influence on body weight as compared to body girth measurements, if they were available. The connection between height measurements and BCS, muscle score, or back fat thickness was also very low or partly negative (Enevoldsen and Kristensen, 1997; Ledinek and Gruber, 2014). Larger animals tended to have a lower body condition. Similar patterns were found for body length in the current study.

### 3.2 Body weight prediction models

Table 4 shows the estimators of the two selected prediction equations Model${}_{\mathrm{HG}\;\mathrm{BG}}$ and Model${}_{\mathrm{HG}\;\mathrm{BG}\;\mathrm{HW}}$. Table 5 includes the P-values and the RMSEs. The fitted curves for the fixed effect physiological stage are shown separately for the lactation and dry periods. The prediction models are only applicable from days -56 to -1 relative to calving in the dry period and from DIM 1 to 365 in lactation. Otherwise, the curvature of fitted curves changes outside these limits.

Within the models with a single body measurement, heart girth was found to be the best body weight predictor (RMSE = 39.0 kg), followed by belly girth (39.3 kg) and hip width (49.9 kg). The RMSE of other linear traits increased from 51.9 kg (body depth) to 57.0 kg (knee width). The usefulness of heart girth due to its connection to body size and body condition was previously reported by Heinrichs et al. (1992, 2017) and Yan et al. (2009). Unlike in previous studies by Ledinek and Gruber (2015) and Stegfellner (2014), BCS predicted body weight with lower accuracy (RMSE = 53.7 kg). Furthermore, the subjectivity of BCS has to be considered. Ferguson et al. (1994) reported that BCS deviated by 0.25 units in 32.6 % and by more than 0.5 units in 9.3 % of the scorings when experienced observers scored the same cow. A lower concordance was found with inexperienced observers (Kleiböhmer et al., 1998). Incorrect scorings also affect predicted values noticeably, due to the high regression coefficient (about 60 kg point${}^{-1}$).

The combination of heart and belly girth in Model${}_{\mathrm{HG}\;\mathrm{BG}}$ reduced the RMSE to 32.5 kg. Including a third body measurement showed Model${}_{\mathrm{HG}\;\mathrm{BG}\;\mathrm{HW}}$ to be the most accurate model (RMSE = 30.4 kg). The prediction accuracy of multiple regressions without body girth traits was even lower than in the models with heart or belly girth as a single predictor. Quadratic effects of body traits did not improve body weight prediction significantly, like in the studies by Yan et al. (2009) and Banos and Coffey (2012). The same was found for more than three body measurements.

Most of the fixed effects genotype, parity, and physiological stage as well as the regression coefficients of all body measurements were significant (P<0.001). Body weight increased degressively with increasing parity and showed the typical simultaneous development of body weight and body measurement of growing animals (Enevoldsen and Kristensen, 1997; Yan et al., 2009). The difference between cows in parity 1 and the oldest parity class was very low, with 9.46 kg in Model${}_{\mathrm{HG}\;\mathrm{BG}\;\mathrm{HW}}$ instead of 88 to 100 kg as reported in other studies (Buckley et al., 2000; Haiger and Knaus, 2010; Blöttner et al., 2011; Ledinek and Gruber, 2015; Ledinek et al., 2018). This shows the strong influence of the additionally included body measurements on body weight in the statistical model.

**Table 5:** Root mean square error (RMSE), P-values, RMSE of the curves fitted to the fixed effect of physiological stage, as well as the root mean square prediction error and the coefficient of determination ($R^{2}$) of the relationship between predicted and observed body weight.

	Model${}_{\mathrm{HG}\;\mathrm{BG}}^{1}$	Model${}_{\mathrm{HG}\;\mathrm{BG}\;\mathrm{HW}}^{1}$
Root mean square error, kg	32.5	30.4
P-values		
Genotype	<0.001	<0.001
Parity	<0.001	<0.001
b_HG${}^{2}$	<0.001	<0.001
b_BG	<0.001	<0.001
b_HW		<0.001
b_HG × genotype	<0.001	<0.001
b_BG × genotype	<0.001	<0.001
b_HW × genotype		0.119
Physiological stage	<0.001	<0.001
RMSE${}^{3}$ curve physiological stage, kg		
lactating	0.73	1.39
dry	4.96	6.76
Validation		
Root mean square prediction error, kg		
lactating	37.0	36.5
dry	41.3	39.9
$R^{2}$ observed${}^{4}$ vs. estimated		
lactating	83.0	83.5
dry	80.1	79.9

${}^{1}$ HG: heart girth; BG: belly girth; HW: hip width; ${}^{2}$ b_: linear regression coefficient; ${}^{3}$ RMSE: root mean square error; ${}^{4}$ $R^{2}$: coefficient of determination of the relationship of observed to predicted body weight.

In the dry period, the fixed effect physiological stage increased from 4.60 to 17.03 kg in Model${}_{\mathrm{HG}\;\mathrm{BG}\;\mathrm{HW}}$. The rising influence can be explained by gestation. During gestation, fetus and fetal membranes themselves regulate nutrient distribution to the conceptus, uterus, and mammary glands. Dairy cows also start replenishing body reserves for the next lactation during the last third of the lactation period (Bauman and Currie, 1980). The growing gravid uterus gains weight, especially in the last third of the gestation period, and therefore in the dry period, with an additional weight of 24 kg on the 190th day of gestation and overall 87 kg on the 285th day of gestation. Fetus accounts for 9.4 and 49.1 kg of this weight, respectively (Bell et al., 1995).

**Figure 1:**
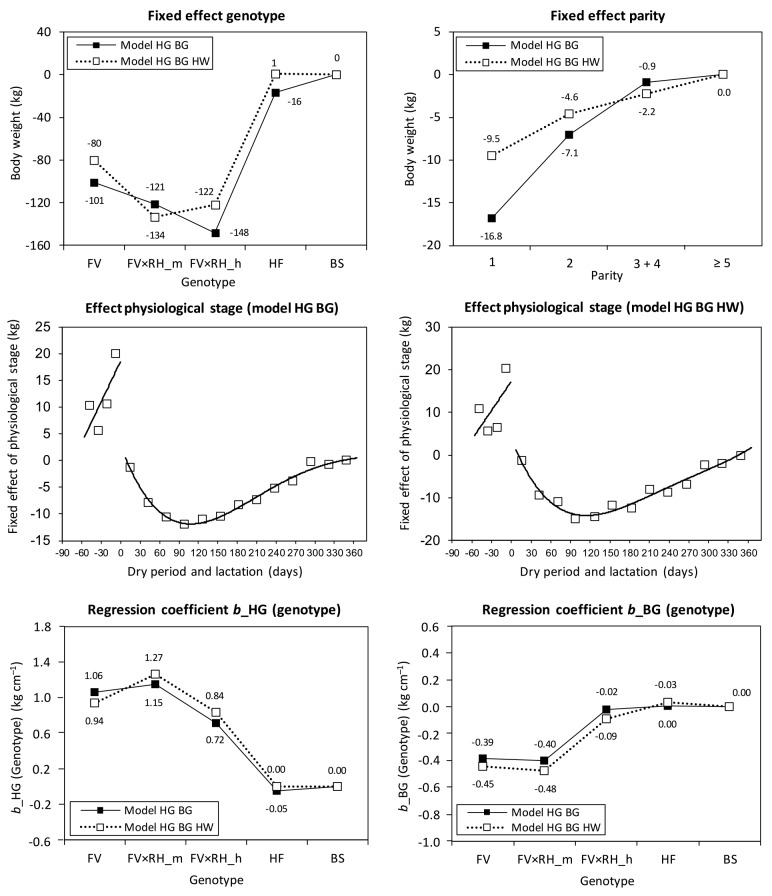
Fixed effect of genotype (FV: Fleckvieh; RH: Red Holstein (gene proportion); HF: Holstein Friesian; BS: Brown Swiss; m: medium; h: high), parity, the curves fitted on the fixed effect of physiological stage, as well as the genotype-specific regression coefficients (b) for heart girth (HG) and belly girth (BG) in the prediction equations Model${}_{\mathrm{HG}\;\mathrm{BG}}$ and Model${}_{\mathrm{HG}\;\mathrm{BG}\;\mathrm{HW}}$ (HW: hip width).

During lactation, a fourth-degree polynomial was necessary to avoid on the one hand the bad fit of the second-degree polynomial, and on the other hand to avoid a premature change in curvature in the third-degree polynomial within the relevant time period. The curve of Model${}_{\mathrm{HG}\;\mathrm{BG}\;\mathrm{HW}}$ showed the typical development during lactation, with the lowest body weight at DIM 114. Therefore, cows reached the nadir of body weight later than in the basic model without body measurements, which again highlights the strong relationship between body weight and body measurements. Belly girth increased continuously during lactation, while BCS and heart girth started to increase later in lactation (data not shown). Andrew et al. (1994) found the lowest body energy content in HF cows at DIM 77, but without a significant change in body weight as compared to other stages of lactation. This indicated that body weight and belly girth depended more on feed intake (gut fill), while heart girth and BCS were connected more to mobilization and recovering of body tissue, as shown by the correlation coefficients. The RMSE of the curves fitted to the fixed effect physiological stage was 6.8 kg during the dry period and 1.4 kg during lactation.

The regression coefficient for heart girth was 2.52 kg cm${}^{-1}$ in Model${}_{\mathrm{HG}\;\mathrm{BG}\;\mathrm{HW}}$ and corresponded to the findings of similar models by Stegfellner (2014, 3.69 kg cm${}^{-1}$) and Yan et al. (2009, 3.09 kg cm${}^{-1}$). The regression coefficient of belly girth was 2.9 kg cm${}^{-1}$ in the current study. It was higher than previously reported, with an average of 1.81, 1.17 and 2.27 kg cm${}^{-1}$ (Yan et al., 2009; Stegfellner, 2014; Ledinek and Gruber, 2015). The influence of hip width in Model${}_{\mathrm{HG}\;\mathrm{BG}\;\mathrm{HW}}$ is quantified with 4.74 kg cm${}^{-1}$. Enevoldsen and Kristensen (1997) examined hip height, hip width, and BCS for body weight prediction. They included a quadratic effect for hip width depending on the data set and model parameters used.

**Figure 2:**
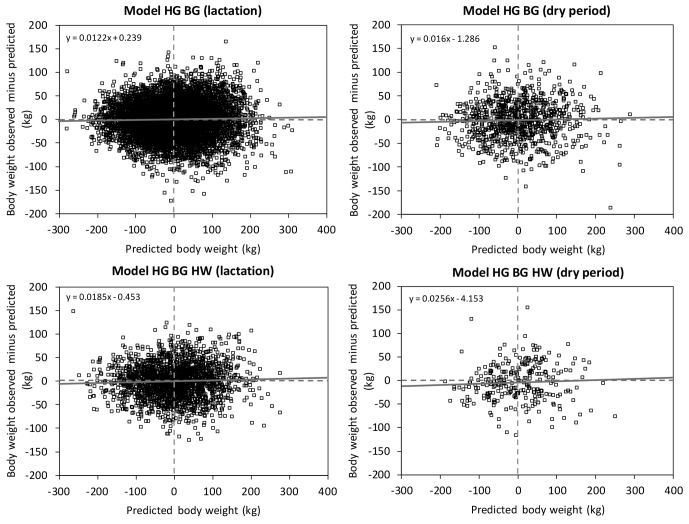
Decomposition of mean square prediction error (Bibby and Toutenburg, 1977) into error of central tendency (ECT, intercept), error of regression (ER, slope), and error of disturbance (ED) shown as a centralized residual plot (St.-Pierre, 2003) of the prediction equations Model${}_{\mathrm{HG}\;\mathrm{BG}}$ and Model${}_{\mathrm{HG}\;\mathrm{BG}\;\mathrm{HW}}$ (HG: heart girth; BG: belly girth; HW: hip width).

The strong influence of body measurements based on genotype became particularly apparent during the testing of the interactions between the fixed effects and regression parameters. To take advantage of the lower RMSE, we additionally introduced genotype-specific regression coefficients (Fig. 1). The genotype-specific regression coefficient of heart girth decreased with increasing RH gene proportion in FV to pure HF from approximately 1 to 0 kg cm${}^{-1}$. In contrast, the genotype-specific regression coefficient of belly girth rose from the negative number range to a slightly positive one in the specialized dairy genotypes (HF, BS, FV × RH_h). The continuous change in traits with increasing gene proportion of specialized dairy breeds in dual-purpose breeds was shown in Ledinek et al. (2018). Body weight is a function of skeletal development (body size), body condition, and gut fill, which depends on milk yield (Yan et al., 2009). The FV groups had a higher BCS, which is strongly correlated with body weight. The dairy types HF and BS had a higher feed intake per kilogram body weight. According to Yan et al. (2009), heart girth was more strongly connected to body weight than belly girth and belly girth was more strongly influenced by gut fill. Therefore, heart girth had a higher influence on body weight in FV than the heart girth of Holstein. In contrast to this, the belly girth of FV had a relatively low influence on the body weight as compared to HF and BS.

### 3.3 Validation of prediction models

**Figure 3:**
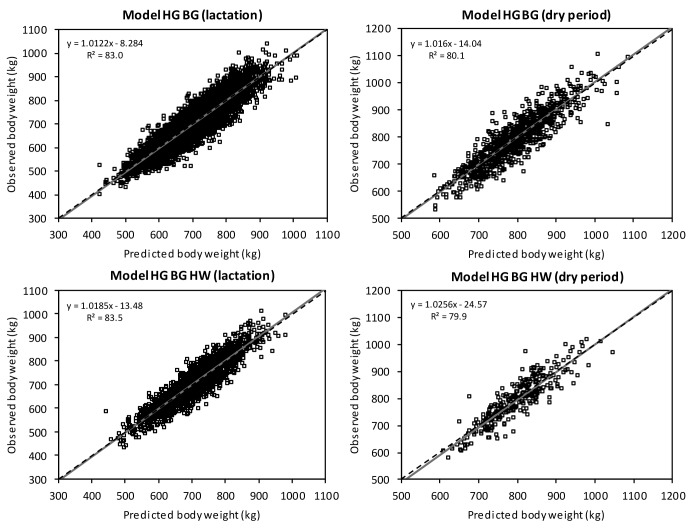
Linear regression of observed on predicted body weight with original values within the prediction equations Model${}_{\mathrm{HG}\;\mathrm{BG}}$ and Model${}_{\mathrm{HG}\;\mathrm{BG}\;\mathrm{HW}}$ (HG: heart girth; BG: belly girth; HW: hip width). If the error of central tendency and regression is 0, then the intercept is 0, the slope is 1, and the regression is identical to the dashed line.

Figures 2 and 3 show the results of the validation of the two body weight equations Model${}_{\mathrm{HG}\;\mathrm{BG}}$ and Model${}_{\mathrm{HG}\;\mathrm{BG}\;\mathrm{HW}}$. The RMSPE and the coefficient of determination ($R^{2}$) of the linear regression of observed body weight on estimated body weight are presented in Table 5 separately for the lactation and dry periods. Table 6 includes the decomposition of the MSPE according to Bibby and Toutenburg (1977) and the regression of the residuals between observed and predicted values on the centered predicted values (St.-Pierre, 2003).

The additional body measurement hip width improved RMSPE from 37.0 to 36.5 during lactation and from 41.3 to 39.9 kg in the dry period. However, it should be considered that Model${}_{\mathrm{HG}\;\mathrm{BG}\;\mathrm{HW}}$ is based on a lower number of data records.

**Table 6:** Decomposition of root mean square prediction error (Bibby and Toutenburg, 1977) as well as the estimators for the regression of the residuals of observed and predicted body weight on the centered predicted body weight (St.-Pierre, 2003).

Model	n (VAL${}^{1}$)	MSPE${}^{2}$	Variance${}^{3}$ caused by				Variance${}^{3}$ (%) caused by				St.-Pierre (2003)	
			ECT	ER	ED		ECT	ER	ED		Intercept	Slope
Model including the body measurements heart girth and belly girth (Model${}_{\mathrm{HG}\;\mathrm{BG}}$)												
lactating	8013	1366	0.057	0.963	1364.5		0.004	0.070	99.925		0.239${}^{*}$	0.0122
dry	872	1709	1.654	1.704	1705.7		0.097	0.099	99.804		$-1.286^{*}$	0.0160${}^{*}$
Model including the body measurements heart girth, belly girth, and hip width (Model${}_{\mathrm{HG}\;\mathrm{BG}\;\mathrm{HW}}$)												
lactating	2080	1331	0.206	2.227	1328.7		0.015	0.167	99.817		$-0.453^{*}$	0.0185${}^{*}$
dry	262	1595	17.25	3.898	1573.4		1.081	0.244	98.673		$-4.153^{*}$	0.0256${}^{*}$

${}^{1}$ VAL: validation; ${}^{2}$ MSPE: mean square prediction error; ${}^{3}$ Bibby and Toutenburg (1977), ECT: error of central tendency; ER: error of regression; ED: error of disturbance; ${}^{*}$ P>0.05.

The $R^{2}$ showed that in the dry period, approximately 80.0 % of variance in the observed values was explained by the prediction models. During lactation, 83.5 % in Model${}_{\mathrm{HG}\;\mathrm{BG}\;\mathrm{HW}}$ was explained. Yan et al. (2009) presented a high adjusted $R^{2}$ of 91 % and a RMSPE of 23.9 kg in their model including heart girth, belly girth, and body length. Similar to the prediction models by Ledinek and Gruber (2015), this high accuracy is the result of data recorded in research herds. Haile-Mariam et al. (2014) validated their body weight prediction model with a 10-fold cross-validation ($R^{2}=47$ %, RMSE = 50 kg). The model included linear traits and BCS, but did not include girth measurements.

The partitioning of the MSPE according to its causes ECT, ER, and ED (Bibby and Toutenburg, 1977) demonstrated that on average, 99.6 % of the variance was caused randomly (Table 6). That means that the models predict body weight without systematic over- and under-estimation. This was visualized in Fig. 2, which shows the intercept and slope of the relationship between the residuals and the centralized predicted values (St.-Pierre, 2003). If no systematic error exists, intercept (ECT) and slope (ER) do not differ significantly from 0 and the linear regression is equal to the x axis. The slope of Model${}_{\mathrm{HG}\;\mathrm{BG}}$ is significant (Table 6), but with 0.012 kg kg${}^{-1}$ of body weight observed, we consider the slope to be negligible. Contrary to this, Fig. 3 shows the deviation of observed to predicted body weight using the original data. Ideally, the linear regression is identical to the 45${}^{\circ }$ line. There are multiple reasons for the lack of a systematic estimation error: the model parameters used describe the systematic causes of variance in body weight comprehensively and the large and heterogeneous data set of a total of 6306 cows facilitated a valid prediction.

## 4 Conclusions

The body measurements with the highest correlation with body weight (heart girth, belly girth, hip width, and body depth) were found to be the best predictors. Body weight prediction based on BCS or solely on linear traits was insufficient, especially by using stature and body length. Therefore, the two body weight prediction equations Model${}_{\mathrm{HG}\;\mathrm{BG}}$ and Model${}_{\mathrm{HG}\;\mathrm{BG}\;\mathrm{HW}}$ were finally chosen. Curves fitted to the fixed effect physiological stage separately for the lactation and dry periods allow a stepless adaption to DIM or the day before calving. The distribution of the MSPE showed that both models predicted body weight without systematic error. Therefore, the chosen model parameters wholly eliminated systematical deviations between predicted and observed values. Furthermore, the large and heterogeneous data set supports a valid prediction. As body weight is an important trait for both management and breeding, the measurement and use of a combination of both heart girth and belly girth are recommended if the use of scales is impossible.

## Supplement

10.5194/aab-61-413-2018-supplement The supplement related to this article is available online at: https://doi.org/10.5194/aab-61-413-2018-supplement.

## Data Availability

The data sets analyzed during the current study are not publicly available as information contained therein could compromise the privacy of third parties.
