# Supplementary material for: Body weight prediction using body size measurements in Fleckvieh, Holstein, and Brown Swiss dairy cows in lactation and dry periods
Source: Arch Anim Breed. 2018 Oct 30;61(4):413–24. doi: 10.5194/aab-61-413-2018 (PMC7065411; doi:10.5194/aab-61-413-2018)
Supplement: The supplement related to this article is available online at: https://doi.org/10.5194/aab-61-413-2018-supplement. [file aab-61-413-supplement.pdf]

Supplement of Arch. Anim. Breed., 61, 413–424, 2018
https://doi.org/10.5194/aab-61-413-2018-supplement
© Author(s) 2018. This work is distributed under
the Creative Commons Attribution 4.0 License.

Open Access
Archives Animal Breeding

Supplement of

Body weight prediction using body size measurements in Fleckvieh, Holstein, and Brown Swiss dairy cows in lactation and dry periods

Leonhard Gruber et al.

Correspondence to: Maria Ledinek (ledinekmaria@yahoo.de)

The copyright of individual parts of the supplement might differ from the CC BY 4.0 License.

Table S1. Description of the estimation and validation subset for Model $_{HG\ BG}$ during lactation (HG = heart girth; BG = belly girth).

Trait	Estimation subset						Validation subset					
	n	Mean	Standard deviation	Variation coefficient	Minimum	Maximum	*n*	Mean	Standard deviation	Variation coefficient	Minimum	Maximum
Body weight, kg	32,116	699	89.7	12.8	400	1,088	8,013	699	89.6	12.8	403	1,042
Body weight estimated, kg							8,013	699	80.6	11.5	422	1,011
Heart girth, cm	32,116	210	10.4	4.9	166	257	8,013	210	10.3	4.9	168	253
Belly girth, cm	32,116	255	13.9	5.5	193	302	8,013	255	13.9	5.5	204	312
Stature, cm	8,439	146	4.5	3.1	128	163	2,122	146	4.7	3.2	130	163
Body length, cm	7,938	90	5.6	6.2	73	110	1,984	91	5.7	6.3	74	111
Pelvis length, cm	8,436	56	3.0	5.3	46	68	2,121	56	3.0	5.4	45	68
Body depth, cm	8,437	84	4.5	5.4	67	99	2,119	84	4.5	5.4	70	99
Hip width, cm	8,435	57	3.4	5.9	45	68	2,119	57	3.4	6.0	46	67
Pin width, cm	8,425	39	4.8	12.2	27	59	2,122	39	4.6	11.8	28	58
Knee width, cm	8,415	54	5.5	10.3	34	71	2,124	53	5.5	10.2	36	69
BCS, points 1–5	31,944	3.16	0.59	18.7	1.00	5.00	7,971	3.16	0.59	18.7	1.00	5.00
Muscle score, points 1–10	31,919	5.1	1.5	29.8	1.0	9.0	7,968	5.1	1.5	29.6	1.0	9.0
Parity	32,116	3.0	2.0	67.2	1	14	8,013	3.0	2.0	67.3	1	13
Day relative to calving	32,116	159	96	60	1	364	8,013	158	97	61	1	364

Table S2. Description of the estimation and validation subset for Model $_{HG\ BG}$ during the dry period (HG = heart girth; BG = belly girth).

Trait	Estimation subset						Validation subset					
	n	Mean	Standard deviation	Variation coefficient	Minimum	Maximum	*n*	Mean	Standard deviation	Variation coefficient	Minimu m	Maximum
Body weight, kg	3,440	794	92.1	11.6	506	1,105	872	794	92.3	11.6	534	1,108
Body weight estimated, kg							872	796	81.5	10.2	586	1,085
Heart girth, cm	3,440	218	11.0	5.1	182	264	872	218	10.9	5.0	188	258
Belly girth, cm	3,440	271	13.2	4.9	227	320	872	271	13.6	5.0	231	314
Stature, cm	909	146	4.7	3.2	131	163	264	145	4.4	3.0	130	156
Body length, cm	846	91	5.9	6.4	75	109	246	92	5.4	5.8	75	105
Pelvis length, cm	909	56	3.1	5.4	48	66	264	56	3.1	5.5	45	66
Body depth, cm	908	86	4.2	4.9	71	99	264	86	4.3	5.0	74	99
Hip width, cm	909	58	3.4	5.8	47	68	262	58	3.3	5.8	48	70
Pin width, cm	907	40	4.9	12.4	28	57	261	40	4.7	11.9	27	56
Knee width, cm	907	55	6.0	10.8	36	70	260	55	6.5	11.8	38	71
BCS, points 1–5	3,429	3.61	0.57	15.9	1.50	5.00	871	3.62	0.58	15.9	1.25	5.00
Muscle score, points 1–10	3,423	5.9	1.5	25.1	1.0	9.0	867	5.9	1.5	25.4	2.0	9.0
Parity	3,440	2.8	1.8	65.4	1	13	872	2.8	1.9	67.3	1	12
Day relative to calving	3,440	–24	15	–61	–56	–1	872	–25	15	–58	–56	–1

Table S3. Number of data records of the body weight prediction models Model $_{HG\ BG}$ and Model $_{HG\ BG\ HW}$ separated for lactation and dry period (fixed effect genotype and parity).

DRY-LAC[1]	EST-VAL[2]	*n*	Genotype[3]					Parity			
			FV	FV×RH_m	FV×RH_h	HF	BS	1	2	3+4	≥ 5
Model including the body measurements heart girth and belly girth (Model $_{HG\ BG}$)											
LAC	EST	32,116	13,457	3,860	1,872	5,171	7,756	9,136	7,100	9,120	6,760
LAC	VAL	8,013	3,330	988	478	1,300	1,917	2,290	1,754	2,288	1,681
DRY	EST	3,440	1,506	423	207	502	802	1009	848	1007	576
DRY	VAL	872	402	93	43	121	213	267	201	250	154
Model including the body measurements heart girth, belly girth, and hip width (Model $_{HG\ BG\ HW}$)											
LAC	EST	8,474	3,530	1,090	495	1,403	1,956	2,292	1,898	2,503	1,781
LAC	VAL	2,080	867	262	119	351	481	564	487	587	442
DRY	EST	909	370	99	46	158	236	274	236	239	160
DRY	VAL	262	104	33	22	36	67	60	79	72	51

[1]DRY = dry period; LAC = lactation

[2]EST = data set for estimation; VAL = data set for validation

[3]FV = Fleckvieh (Red Holstein genes ≤ 10.0 %); RH = Red Holstein; HF = Holstein Friesian; m = medium proportion of RH genes (> 10.0 – ≤ 44.5 %); h = high proportion of RH genes (> 44.5 %); BS = Brown Swiss

Table S4. Number of data records of the body weight prediction models Model $_{\text{HG BG}}$ and Model $_{\text{HG BG HW}}$ separated for lactation and dry period (fixed effect of physiological stage: lactation and dry period).

DRY-LAC[1]	EST-VAL[2]	Dry period (weeks ante partum)				Lactation (months post partum, 28 days per month)												
		7–8	5–6	3–4	1–2	1	2	3	4	5	6	7	8	9	10	11	12	13
Model including the body measurements heart girth and belly girth (Model $_{\text{HG BG}}$)																		
LAC	EST					2,950	3,054	2,944	2,909	2,888	2,695	2,765	2,618	2,644	2,587	2,020	1,284	758
LAC	VAL					791	777	690	730	659	732	715	670	644	543	493	346	223
DRY	EST	483	860	1,028	1,069													
DRY	VAL	131	237	272	232													
Model including the body measurements heart girth, belly girth, and hip width (Model $_{\text{HG BG HW}}$)																		
LAC	EST					685	844	773	828	760	726	739	662	685	635	553	367	217
LAC	VAL					153	225	185	169	180	180	177	170	158	174	149	109	51
DRY	EST	131	273	290	215													
DRY	VAL	46	75	94	47													

[1]DRY = dry period; LAC = lactation

[2]EST = data set for estimation; VAL = data set for validation
